# Bacterial carrageenases: an overview of production and biotechnological applications

**DOI:** 10.1007/s13205-016-0461-3

**Published:** 2016-06-23

**Authors:** Prakram Singh Chauhan, Arunika Saxena

**Affiliations:** 1 Faculty of Pharmacy and Pharmaceutical Sciences, Monash University Parkville Campus, 381, Royal Parade, Melbourne, VIC 3052 Australia; 2 Department of Chemistry, Samrat Prithviraj Chauhan Government College, Beawar Road, Ajmer, Rajasthan India

**Keywords:** Carrageenan, Bacterial carrageenase, Glycoside hydrolase, Biotechnological applications

## Abstract

Carrageenan, one of the phycocolloids is a sulfated galactan made up of linear chains of galactose and 3,6-anhydrogalactose with alternating α-(1 → 3) and β-(1 → 4) linkages and further classified based on the number and the position of sulfated ester(s); κ-, ι- and λ-carrageenan. Enzymes which degrade carrageenans are called k-, ι-, and λ-carrageenases. They all are endohydrolases that cleave the internal β-(1–4) linkages of carrageenans yielding products of the oligo-carrageenans. These enzymes are produced only by bacteria specifically gram negative bacteria. Majority of the marine bacteria produce these enzymes extracellularly and their activity is in wide range of temperature. They have found potential applications in biomedical field, bioethanol production, textile industry, as a detergent additive and for isolation of protoplast of algae etc. A comprehensive information shall be helpful for the effective understanding and application of these enzymes. In this review exhaustive information of bacterial carrageenases reported till date has been done. All the aspects like sources, production conditions, characterization, cloning and- biotechnological applications are summarized.

## Introduction

Hydrocolloids can be defined as substances that interact with water to form colloid systems. Hydrocolloid polysaccharides (agar, alginates and carrageenans) have significant importance, both technologically and economically, since they are used in the various biotechnological industries due to their distinct physico-chemical properties (Knudsen et al. 2015; Zhu and Ning 2016; Xiao et al. 2016).

Carrageenans are commercially important hydrophilic colloids (water soluble gums) which occur as matrix material in numerous species of red seaweeds [comprise up to 50 % dry weight (Rhodophyta)] wherein they serve a structural function analogous to that of cellulose in land plants. They are a group of biomolecules composed of linear polysaccharide chains with sulphate half-esters attached to the sugar unit. It exists in different forms depending on the number of sulphate substituents per disaccharide unit: one in κ-carrageenan, two in ι-carrageenan and three in λ-carrageenan. These properties allow carrageenans to dissolve in water, form highly viscous solutions, and remain stable over a wide pH range (Ruiter and Rudolph 1997; Yao et al. 2014; Liu et al. 2016).

Microbial enzymes which hydrolyze hydrocolloids have drawn considerable interest recently because enzymatic degraded products of carrageenan is still in infancy compared to that of other hydrocolloids such as agar, alginate etc. Enzymes which degrade carrageenans are called k-, ι-, and λ-carrageenases. They all are endohydrolases that cleave the internal β-(1–4) linkages of carrageenans yielding products of the oligo-carrageenans. Oligo-carrageenan produced by the action of microbial enzymes can be more advantageous than produced by acid hydrolysis because enzymes are highly specific to their substrates and they generate oligo-derivatives are uniform in molecular weights (Yao et al. 2014).

The oligosaccharides derived from carrageenan have been shown to exhibit antitumor, anti viral activity which indicated that oligo-carrageenan could possess significant potential for biomedical and physiological applications (Li et al. 2014a, b). In addition to this carrageenase have a various important application such as bioethanol production, textile industry, as a detergent additive and for isolation of protoplast of algae etc.

Due to their versatile industrial applicability, interest has greatly increased in carrageenases during the past decade (Necas and Bartosikova 2013). A number of carrageenases from various organisms are being reported, cloned and studied at molecular level. In this review, information on carrageenases from most of the microorganisms currently reported have been compiled in terms of the production conditions, enzyme properties, gene cloning and potential industrial applications.

## Carrageenan occurrence and structure

Majority of the seaweeds that produce carrageenan as their main cell-wall material belong to the red algae, or Rhodophyta. The carrageenans are extracted from the carrageenophyte red seaweed genera *Kappaphycus*, *Gigartina*, *Eucheuma*, *Chondrus*, and *Hypnea*, in which the carrageenans comprise up to 50 % of the dry weight (Knudsen et al. 2015). κ-Carrageenan is mostly extracted from *Kappaphycus alvarezii*, known in the trade as *Eucheuma cottonii*, while ι-carrageeman is predominantly produced from *Eucheuma denticulatum*, also known as *Eucheuma spinosum.* λ-Carrageenan is obtained from seaweeds within the *Gigartina* and *Chondrus* genera, which as sporophytic plants produce λ-carrageenan while they make a κ/ι-hybrid as gametophytic plants (Van De Velde et al. 2001). These κ/ι -hybrid type carrageenans, also known as ‘‘kappa-2” or "weak-gelling kappa carrageenans”, consist of mixed polysaccharide chains containing both κ- and ι -units and range from almost pure ι-carrageenan to almost pure κ-carrageenan (Table 1).

**Table 1:** Chemical structure and properties of different carrageenans

Polysaccharide	Sources	Backbone made up of alternating units		No. of sulfate ester	Properties		
		A	B		Solubility	Gel formation	Viscosity
κ-Carrageenan	*Kappaphycus alvarezii*	3,6-Anhydro-d-galactose	d-Galactose 4-sulfate	One	Hot solution	KCl promote gelling	Low
ι-Carrageenan	*Eucheuma denticulatum*	3,6-Anhydro-d-galactose-2-sulfate	d-Galactose 4-sulfate	Two	Soluble in cold or hot aqueous solution	Ca^2+^ promote gelling	High
λ-Carrageenan	*Gigartina* and *Chondrus* genera	d-galactose-2,6-disulfate	d-galactose, d-galactose-2-sulfate	Three	Soluble in cold or hot aqueous solution	Non-gelling	High/medium

The seaweed is dried quickly to prevent degradation, and is then baled for shipment to processing facilities. The seaweed is repeatedly washed to remove gross impurities such as sand, salt, and marine life, and then undergoes a hot alkali extraction process, releasing the CG from the cell. Once CG is in a hot solution, it undergoes clarification and then is converted to powder (Rowe et al. 2009). Meanwhile, extraction parameters (such as temperature, pH, duration) and alkaline pre-treatment duration have important effects on the chemical structure and gelling properties (Hilliou et al. 2006).

**Fig. 1:**
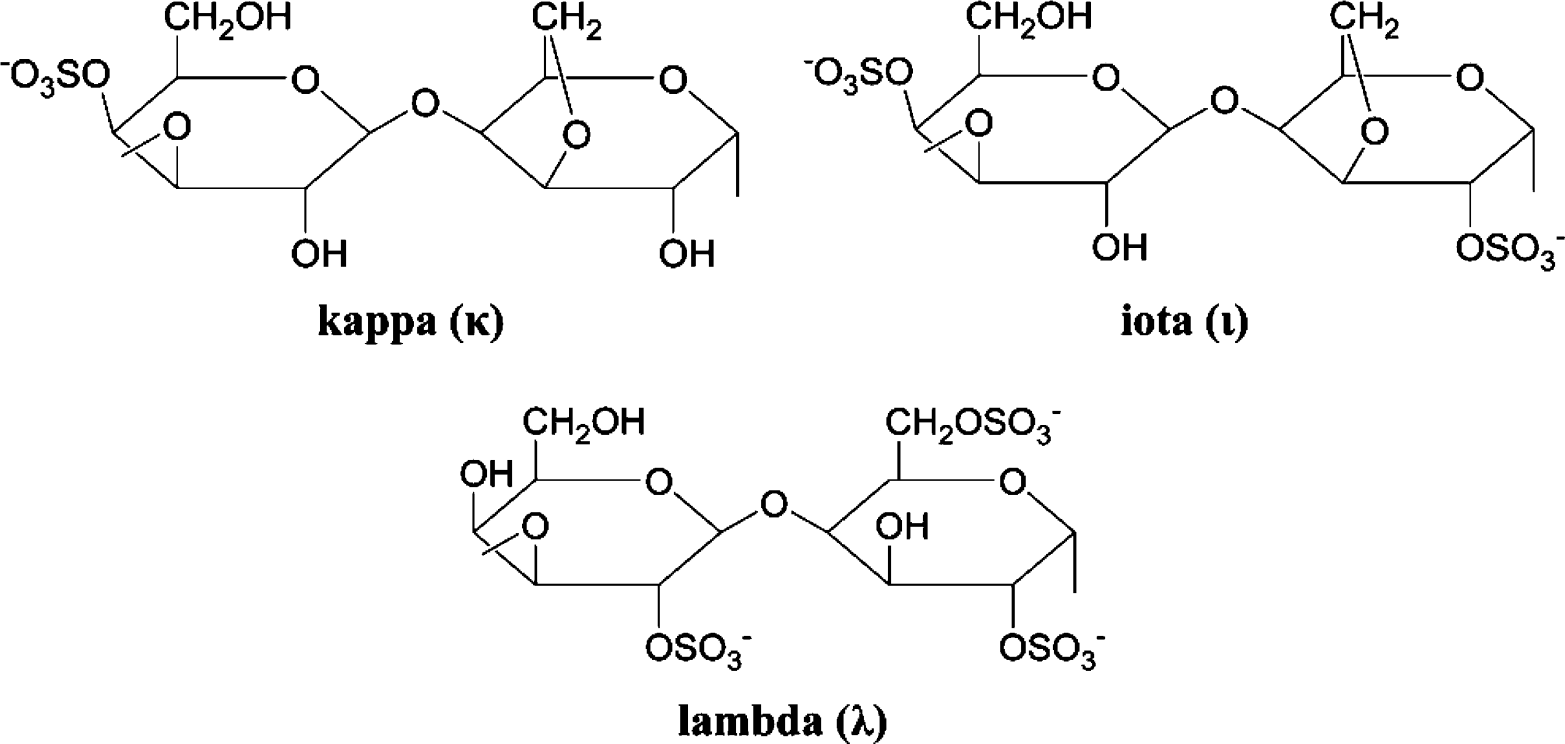
Structure of kappa, iota and lambda carrageenans

Carrageenan is a high molecular mass material (>100 kDa) with a high degree of polydispersity. Carrageenans are hydrophilic sulfated linear galactans that mainly consist of d-galactose and 3,6-Anhydro-d-galactose (3,6-AG) units bound together with alternating α-1,3 and β-1,4 linkages. The 3-linked units occur as the 2-and 4-sulfate, or unsulfated, while the 4-linked units occur as the 2-sulfate, the 2,6-disulfate, the 3,6-anhydride, and the 3,6-anhydride-2-sulfate. Sulfation at C3 apparently never occurs. Except the galactose and sulfate, other carbohydrate residues (for example xylose, glucose and uronic acids) and substituents (for example methyl ethers and pyruvate groups) are present in carrageenans (Michel et al. 1997; Knudsen et al. 2015) (Fig. 1).

This base structure is consistent in the three main commercially used carrageenans, κ-, ι-, and λ-carrageenan. κ-Carrageenan has one sulfate ester, while ι-and λ-carrageenan contain two and three sulfates per dimer, respectively (Fig. 1). The number and position of ester sulfate groups as well as the content of 3.6-AG influence the properties of different carrageenans. Higher levels of ester sulfate mean lower solubility temperature and lower gel strength. Kappa type carrageenan has an ester sulfate content of about 25–30 % and a 3,6-AG content of about 28–35 %. Iota type carrageenan has an ester sulfate content of about 28–30 % and a 3,6-AG content of about 25–30 %. Lambda type carrageenan has an ester sulfate content of about 32–39 % and no content of 3,6-AG. Considering that each natural carrageenan is a complex galactose-based polysaccharide that has different quantities of sulphate esters at different positions and with different distributions, the term disaccharide repeating unit refers to the idealized structure (Campo et al. 2009).

## Microbial carrageenanolytic system

### Enzymatic hydrolysis of carrageenan

**Fig. 2:**
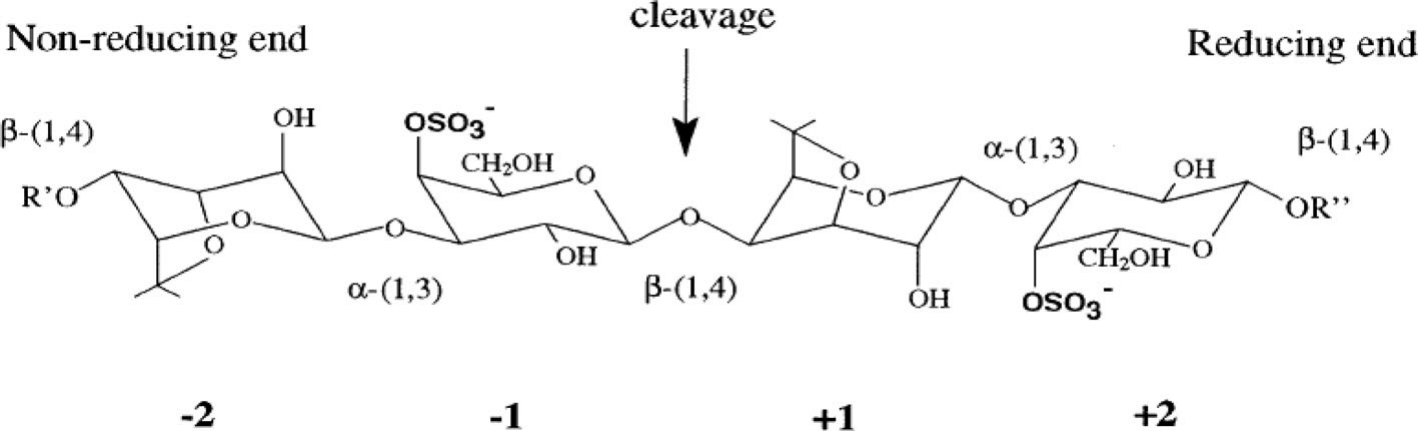
Schematic representation of two disaccharide-repeating units of κ-carrageenan having reducing as well as non reducing ends. Carrageenan cleavage site are indicated by *arrow* (Michel et al. 2001a)

Due to the complex chemical structure of carrageenan and specific cleavage of linkages in the backbones of carrageenan polymers, specific enzymes are required for the respective structures without the risk of modification of the native structure. Enzymes, which degrade carrageenans, are called κ-carrageenases (EC 3.2.1.83), ι-carrageenases (EC 3.2.1.157), and λ-carrageenases (EC 3.2.1.162). They all are endohydrolases that cleave the internal β-(1-4) linkages of carrageenans yielding oligogalactans of either neocarrabiose or neoagarobiose series (Fig. 2) (Barbeyron et al. 2000). It was reported that κ-carrageenases and ι-carrageenases degrades carrageenan by hydrolyzing the β-(1-4) [breaking internal linkages rather than hydrolyzing units from the ends] linkages to a series of homologous, even-numbered oligosaccharides. Although, both enzymes are processive, in which the enzyme does not dissociate from the substrate and instead slides along the polysaccharide, cleaving all possible bonds (DP4s and DP2s). In contrast, λ-carrageenase cleaves internal linkages more randomly manner, resulting in higher amounts of DP6s (and possible other higher DPs as products) compared to the products from κ- and ι-carrageenase hydrolysis. Since these hydrolases display strict substrate specificity, they obviously recognize the sulfation pattern on the digalactose repeating unit. Digestion by carrageenases generates oligo-galactans of various sizes, most likely carbohydrates with a degree of polymerization (DP) of 2, 4, and 6. The reason for the production of different DPs is a result of the heterogenous carrageenan structure and the mechanisms that the enzymes follow. The alternating α-1,3 and β-1,4 linkages in the carrageenans results in successive β-1,4 linkages to be in opposite orientations and hence only every second disaccharide is in the right position for cleavage (Michel et al. 2001a; Guibet et al. 2007; Lemoine et al. 2009). A chain length of four sugar residues is required for the binding of carrageenases to ensure hydrolysis. The substrate binding surface can be split into different subsites. Subsites are numbered from −*n* to +*n* (*n* being an integer) from nonreducing to reducing ends of the mannan substrate, respectively (Davies et al. 1997). Cleavage of the glycosidic bond occurs between subsite +1 and −1 (Fig. 2). Oligo-carrageenan produced by the action of microbial enzymes can be more advantageous than produced by acid hydrolysis because enzymes are highly specific to their substrates and they generate oligo-derivatives are uniform in molecular weights (Yao et al. 2014).

### Reaction mechanism of carrageenan degrading enzymes

**Fig. 3:**
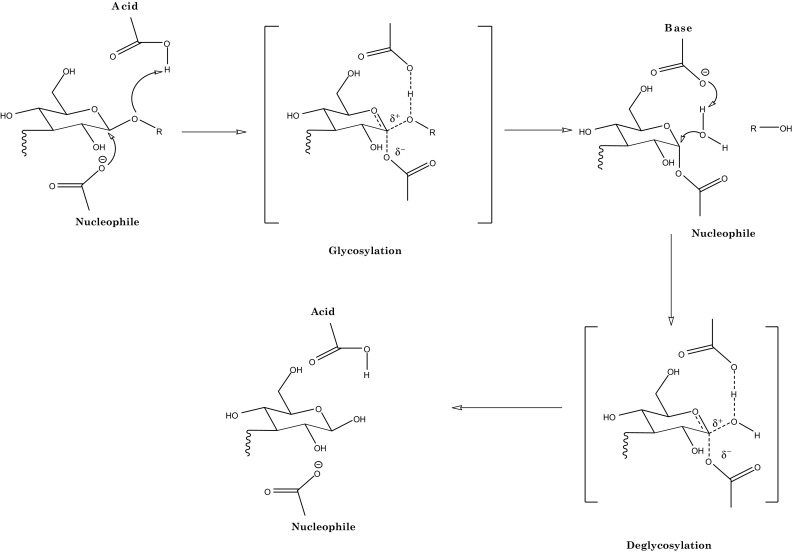
Retaining mechanism for the degradation of carrageenan by κ-carrageenases via double displacement reaction (Chauhan et al. 2012)

Different carrageenases exhibit different mechanism for the hydrolysis polymer. κ-Carrageenases hydrolyse their substrates by a retaining mechanism which occurs via double-displacement reaction (Fig. 3) (Chauhan et al. 2012, 2014a; Chauhan and Gupta 2016). The key amino acids involved in the retaining mechanism reaction are two catalytic carboxylate residues that exist at opposite sides of sugar plane. The enzymes in this mechanism works in tandem involving two steps i.e. glycosylation and deglycosylation (Chauhan et al. 2015). In the first step of double displacement mechanism, it facilitates the departure of leaving group by donating proton to the glycosyl oxygen atom. Simultaneously a second carboxylate moiety facilitates the nucleophilic attack on the anomeric carbon to form glycosyl-enzyme intermediate (Fig. 3). In the second step, the deprotonated acid/base functions as a general base which activates nucleophile. This activated nucleophile then cleaves the glycosyl enzyme complex (Fig. 3). In spite of wide array of GH, hydrolysis of glycosidic bond is generally catalyzed by either glutamate (Glu) or Aspartate (Asp).

**Fig. 4:**
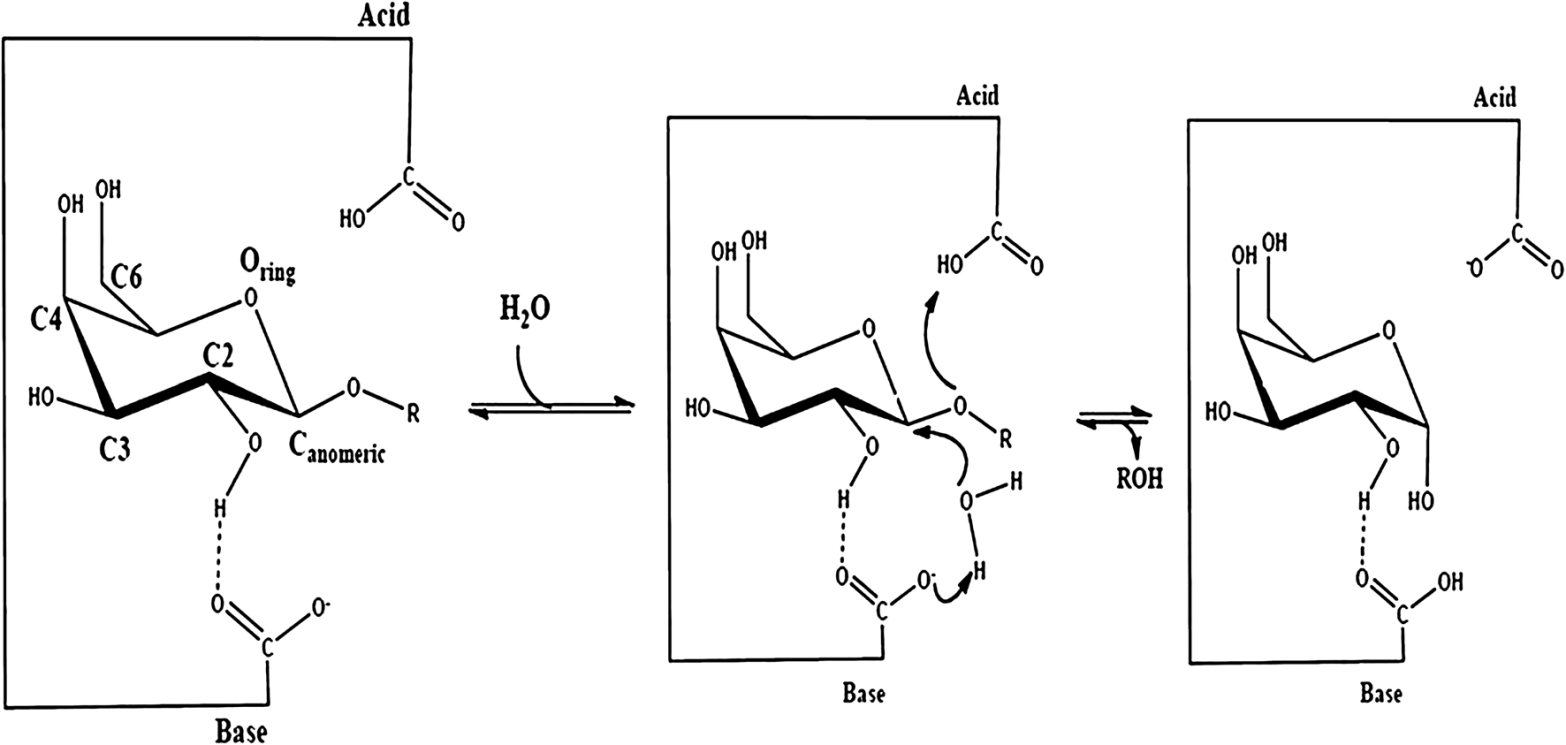
Inverting mechanism for the degradation of carrageenan by ι-carrageenases via single displacement reaction (Bras et al. 2012)

ι-Carrageenases hydrolyse their substrate by inverting mechanism (Fig. 4), In this, there is an inversion of the anomeric configuration of the starting material. Here, the two crucial carboxylic residues act as general acid and base catalysts and these groups are *circa* 10.5 Å apart from each other. It this specific case, this distance is larger than in retaining GHs because the substrate and the water molecule must be present simultaneously in the active site of the enzyme during the hydrolytic process. Figure 4 shows the proposed mechanism of action for inverting GHs, which occurs via a single-displacement type of mechanism. In this case, one of the carboxylate residues protonates the scissile glycosidic oxygen atom while the other coordinates the nucleophile (i.e., the water molecule) to assist its deprotonation and in this way completes the hydrolysis reaction (Michel et al. 2001b, 2003).

### Assay methods for carrageenases

Numerous screening methods exist for detecting carrageenanolytic activity in microorganisms. A solid medium giving fast assays is useful for the direct measurement and isolation of carrageenan degrading organisms from natural substrates. Common screening techniques used for the detection of carrageenan degrading enzymes involve plate assays where the individual polymer, is added into the basal growth medium. The formation of analogous hydrolases is indicated by the plate depression-forming activity or liquefaction or clearing of the opaque medium as the substrate is hydrolyzed by the enzymes formed by the growing colonies (Henares et al. 2010). In addition to this by flooding the solid medium with 10 % cetylpyridinium chloride, the colonies with clear zones against a white background is another way to find out carrageenan-degrading microorganisms (Ohta and Hatada 2006). Similar to this enzyme is kept in a well cut in an agar medium containing a carrageenan substrate. The agar surface commonly swaps with a Lugol’s solution (Potassium iodide and Iodine) to develop the zones of enzyme activity near the cup. The width of the cleared zone is quantify with calipers and recorded. Width is converted to enzyme activity using a dilution of the enzyme and plotting zone width with respect to the log of enzyme concentration (Kang and Kim 2015).

In liquid assay method carrageenase activity is determined using carrageenan as a substrate. In viscometric method, flow time of the digest (enzyme + substrate) was determined at once and also at regular intervals after putting the viscometer in water bath at particular temperature. One viscometric unit of enzyme activity was defined as the amount of enzyme which would reduce the specific viscosity of the substrate by 50 % (Mclean and Williamson 1979).

In spectrophotometric method reducing sugar released from the substrate by the action of enzyme was determined by Somogyi–Nelson procedure (Somogyi 1952). Here addition of alkaline copper sulfate was added which helpful to stop the reaction between enzyme and substrate. In Dinitrosalicylic acid (DNSA) method reducing sugar released by the action of enzyme on substrate was quantified with Dinitrosalicylic acid (DNSA) reagent (Miller 1959) by taking the absorbance at 560 nm whereas in Kidby method reaction mix were incubate with Kidby solution (1 % Na_2_CO_3_ and 0.03 % potassium hexacyanoferrate III). Color development was achieved by placing tubes in a water bath 100 °C. Finally, the spectral absorbance of samples at 420 nm was measured (Kidby and Davidson 1973). One unit of κ-carrageenase activity was defined as the amount of enzyme needed to release 1 μmol reducing sugars (d-galactose equivalent) per min.

In neocuproine method, assay mixture was incubated for particular time and temperature followed by the addition of 1 ml of alkaline copper reagent (4 % Na_2_CO_3_, 1.6 % Glycine and 0.045 % CuSO_4_) to stop the reaction. After that 1 ml of 5 mM Neocuproine–HCl reagent was added to the tube and was kept in a boiling water bath. It was necessary to add ethanol (50 % v/v) to the reaction mixture before measuring the absorbance. One unit of the enzyme activity was defined as the amount which liberated 1 μmol galactose equivalent per minute under the assay conditions (Dygert et al. 1965).

## Carrageenases classification and structural characteristics

Based on amino acid sequences, these enzymes belong to distinct glycoside hydrolase (GH) families. Although the various carrageenans are chemically rather close, but the respective GHs that degrade different types of carrageenans do not belong to the same family. κ-Carrageenases belong to GH16 (family 16 of the GHs) (Barbeyron et al. 1994, 1998), a polyspecific family which encompasses at least eight different enzymatic activities, including β-agarases ((http://www.cazy.org/) (Coutinho and Henrissat 1999; Cantarel et al. 2009). Phylogenetic analysis investigations demonstrated that family GH16 enzymes have evolved from a common ancestor and that κ-carrageenases have likely emerged from the β-agarase branch (Jam et al. 2005) and they belong to clan GH-B. κ-Carrageenases hydrolyse β-(1 → 4) glycosidic linkages with retention of the anomeric configuration (Potin et al. 1995). ι-Carrageenases define the unrelated family GH82 (Michel et al. 2006; Rebuffet et al. 2010), while λ-carrageenases constitute a new GH family, although it has not been yet classified in the carbohydrate-active enzymes (CAZy) database (Barbeyron et al. 1998). The κ-carrageenase belongs to this family adopts a typical β-jellyroll fold (polypeptide chain is wrapped around a barrel core like a jelly roll) whereas ι-carrageenases carry β-helix in 3D-structure (Guibet et al. 2007).

Carrageenases have been poorly characterized from a structural point of view. Till now three dimensional structure of only two carrageenase i.e., κ-carrageenase (CgkA) from *Pseudoalteromonas carrageenovora* and ι-carrageenase (CgiA_Af) from *Alteromonas fortis* has been solved. Both of the enzymes displays a tunnel-shaped active site suggesting a processive mode of action in which the enzyme does not dissociate from the substrate and instead slides along the polysaccharide, cleaving all possible bonds. The κ-carrageenan chain is composed of alternating neutral and negatively charged sugars (DA and G4S, respectively). To accommodate the dual nature of its substrate, κ-carrageenase features in its active site both conserved aromatic and basic residues which are predicted to interact with DA and G4S moieties, respectively (Michel et al. 2001b) whereas carrageenan which consists of only negatively charged sugars (DA2S and G4S) is recognized by κ- and ι-carrageenase essentially through ionic interactions between its sulfate groups and several conserved arginine residues of the protein (Michel et al. 2003). Studies on κ- and ι-carrageenases have provided some insight into sulfated polysaccharide–protein interactions; but the chemical complexity of sulfated polysaccharides is a patent obstacle to such analyses and researcher looking for better structural characterization (Michel et al. 2001a).

**Fig. 5:**
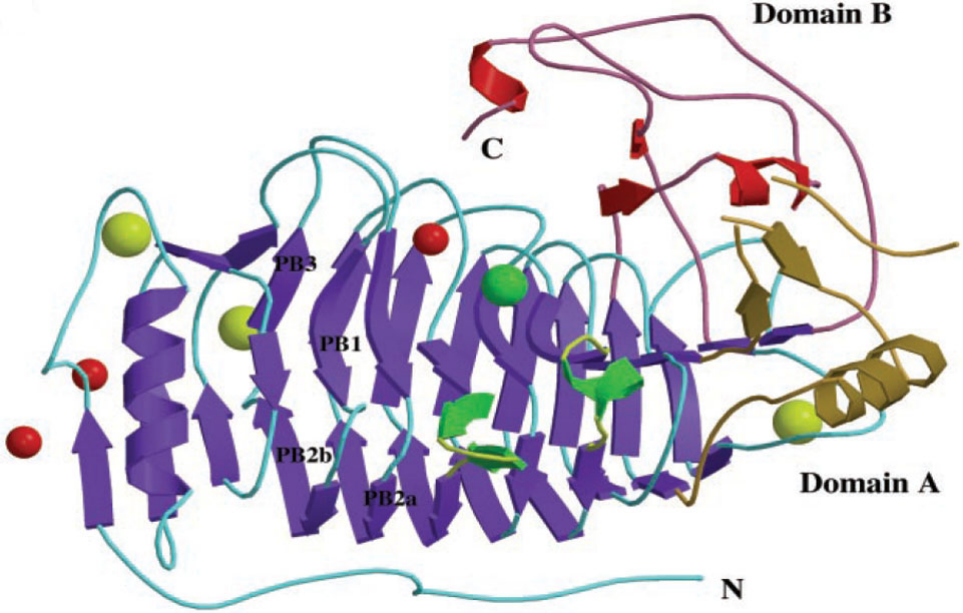
Three dimensional structure of CgiA_Af carrageenase from *Alteromonas fortis*. The enzyme assembly contains domains, antiparallel sheet, helix and ions which are represented with different color (Michel et al. 2001b). Domain A (*Gold*), Domain B (*Red*) and helix core (*Blue*) are structural domains containing mainly β-sheets contains the catalytic center. Small T1 extension, containing an antiparallel sheet (β16-β17) and α-helix (α2), is shown in* green*. Sodium, calcium, and chloride ions are represented by* red*,* yellow*, and* green* spheres, respectively involved in stabilizing the enzyme structure

However, they all hydrolyze carrageenan substrates, but these carrageenases do not share significant sequence homology. Although they share some common binding site for ions which helpful in stabilizing the enzyme. Here the three dimensional structure of ι-carrageenase (CgiA_Af) from *Alteromonas fortis* is shown in Fig. 5. The enzyme folds into a right-handed parallel β-helix of 10 complete turns with flanked by two domains (A and B) in the C-terminal region. Glu245, Asp247, or Glu310, in the cleft of the enzyme, are proposed as candidate catalytic residues. The protein contains one sodium and one chloride binding site and three calcium binding sites shown to be involved in stabilizing the enzyme structure. The crystallographic structure of this enzyme was also solved in the presence of substrate oligocarrageenans, where a tetrasaccharide and a disaccharide have been located in subsites +4 to +1 and −3 to −4, respectively (Michel et al. 2001b). The *A. fortis* iotase hydrolyzes the β-(1–4) bond by an inverting mechanism and produces neo-ι-carratetraose and neo-ι-carrahexaose as end products. Like the ι-carrageenases, κ-carrageenases are endohydrolases, breaking internal linkages rather than hydrolyzing units from the ends. Also, both enzymes are processive, hydrolyzing several units in succession. In contrast, λ-carrageenase cleaves internal linkages randomly (Michel et al. 2003; Guibet et al. 2007; Knudsen et al. 2015).

## Sources of carrageenases

Carrageenases have been produced from marine bacteria that belong to two distantly related lineages, *Proteobacteria* and *Bacteroidetes*, although most of the isolates belong to the former group. Carrageenases appears to carry out different functions, depending on the producing organism. Carrageenases from bacteria are often employed in the degradation of polysaccharides particularly carrageenan from marine rhodophytes, are major raw materials for a number of industries worldwide. In this regard, studies on important carrageenan degraders reported in the recent years are listed in Table 2. Among bacteria, degradation is mostly confined to Gram negative, mainly various *Pseudoalteromonas, Cellulophaga, Pseudomonas, Cytophaga, Tamlana, Vibrio, Catenovulum, Microbulbifer, Zobellia, Alteromonas* (Shangyong et al. 2013; Yao et al. 2013; Ziayoddin et al. 2014; Mou et al. 2004; Feixue et al. 2010; Li et al. 2015; Hatada et al. 2011; Liu et al. 2013; Michel et al. 2001b; Zhu and Ning 2016). However, some Gram positive bacteria like *Bacillus* sp. and *Cellulosimicrobium* have also been reported to produce carrageenase (Kang and Kim 2015; Youssef et al. 2012). Till date no fungal species is identified which are able to produce carrageenase which is going to be very hot area in near future for exploring the microbial diversity.

**Table 2:** Production conditions and characteristics of bacterial carrageenase from different microorganisms

S. no.	Name of organism	Carbon source/fermentation conditions	Temp. optima (°C) of activity	Temp. stability	pH optima of activity	pH stability	Molecular weight of protein (KDa)	References
1	ALAB-001	YE^a^ and tryptone in seawater/30 °C/8 days	NR^n^	NR^n^	NR	NR^n^	NR^n^	Tayco et al. (2013)
2	*Bacterium* 1	WY^b^ medium having Iota-carrageenan/22 °C/150 rpm/12 h	40	NR^n^	8.0	NR^n^	NR^n^	Greer and Yaphe (1984)
3	*Bacterium* 1	MM^c^ having carrageenan/22 °C/48 h/pH 7.5	30	NR^n^	7.2	NR^n^	NR^n^	Bellion et al. (1982)
4	*Bacillus* sp. Lc50-1	NB^d^/55 °C	75	50 %/75 °C/45 min	8.0	70 %/pH 6–9/15 min/75 °C	37 kDa	Li et al. (2014 b)
5	*Bacillus* sp. SYR4	RSPM^e^/37 °C/180 rpm/14 days/pH 7.4	30	NR^n^	7.5	NR^n^	NR^n^	Kang and Kim (2015)
6	*Catenovulum* sp. LP	LM^f^/30 °C/48 h/pH 7.5	35	50 %/35 °C/36 h	6.0	>50 %/pH 5.0–9.0/35 °C/1 h	75.5 kDa	Li et al. (2015)
7	*Cellulosimicrobium cellulans*	Kappa-carrageenan/37 °C/250 rpm/24 h/pH 7.5	30	NR^n^	6.0	NR^n^	NR^n^	Beltagy et al. (2012)
8	*Cellulosimicrobium cellulans*	ZM^g^/37 °C/250 rpm/24 h/pH 7.5	37	NR^n^	7.5	NR^n^	NR^n^	Youssef et al. (2012)
9	*Cellulophaga lytica* strain N5-2	Kappa-carrageenan/35 °C/20 h	35	>85 %/40 °C/pH 7.0/150 min	7.0	>80 %/pH 7.0/360 min/35 °C	40.8	Yao et al. (2013)
10	*Cellulophaga* sp. QY3	Iota-carrageenan/25 °C/150 rpm/20 h/pH 7.0	50	>80 %/50 °C/pH 7.0/60 min	7.0	>70 %/pH 5.0–10.6/720 min/4 °C	48.3 kDa	Ma et al. (2013)
11	*Cytophaga* lk-C783	ZM^g^ having carrageenan/25 °C/150 rpm/72 h	25 °C	50 %/50 °C/pH 7.0/10 min	7.6	NR^n^	100 kDa	Sarwar et al. (1983a, b, 1987)
12	*Cytophaga* MCA-2	ZM^g^ having carrageenan/32 °C/150 rpm/36 h/pH 7.5	28	0 %/55 °C/30 min	7.2	32 %/pH 10.83/24 h	30 kDa	Mou et al. (2004)
13	Dsij strain	ZM^g^/22 °C/250 rpm/24–40 h	40	100 %/30 °C/pH 7.2/120 min	7.2	100 %/7.0/60 min/40 °C	40 kDa	Potin et al. (1991)
14	Marine bacteria	Carrageenan/20 °C–25 °C/100 rpm/72–96 h	40	NR^n^	7.5	NR^n^	NR^n^	Yaphe and Baxter (1955)
15	*Pseudoalteromonas* sp. QY203	Kappa-carrageenan/25 °C/150 rpm/48 h	45	70 %/35 °C/pH 7.2/48 h	7.2	>70 %/6.5–9.0/6 h/4 °C	34	Shangyong et al. (2013)
16	*Pseudomonas aeruginosa* ZSL-2	Kappa-carrageenan + MMSM^h^/37 °C/180 rpm/24 h	28	NR^n^	8.0	NR^n^	NR^n^	Ziayoddin et al. (2014)
17	*Pseudoalteromonas* strain CL19	Kappa-carrageenan/20 °C/72 h	35	NR^n^	7.0	NR^n^	100 kDa	Ohta and Hatada (2006)
18	*Pseudomonas carrageenovora* HLX 250	Carrageenan/25 °C/60-66 h/pH 7.02	30	100 %/40 °C/210 min	7.5	NR^n^	NR^n^	Weigl and Yaphe (1966)
19	*Pseudoalteromonas carrageenovora* IFO 12985	BM^i^ having kappa or Iota-carrageenan/27 °C/48 h	NR^n^	NR^n^	NR^n^	NR^n^	1.4 kDa	Henares et al. 2010)
20	*Pseudomonas elongata* MTCC 5168	LM^f^ having carrageenan/37 °C/180 rpm/24–32 h	40	>90 %/−20 °C/25 days	5.6/7.7	NR^n^	128 kDa	Khambhaty et al. 2007b)
21	*Pseudoalteromonas porphyrae* LL-1	2216E medium having carrageenan/28 °C/160 rpm/28 h/pH 7.5	55	>95 %/30 °C/1 h	8.0	100 %/pH 8.0/6 h/4 °C	40 kDa	Liu et al. 2011)
22	*Pseudoalteromonas* WZUC10	BM^j^/25 °C/200 rpm/48 h/pH 7.5	30	90 %/40 °C/pH 7.1/2 h	7.5	NR^n^	45 kDa	Zhou et al. 2008)
23	*Pseudoalteromonas carrageenovora* ATCC-43555	Y2MM^k^ having carrageenan/20 °C/48 h/250 rpm	30	NR^n^	7.5	NR^n^	97 kDa	Guibet et al. 2007)
24	*Pseudomonas carrageenovora* NCMB no. 302	LM^f^ having carrageenan/25 °C/100 rpm/60–80 h	40	NR^n^	8.0	NR^n^	35	Mclean and Williamson 1979; Ostgaard et al., (1993); Dyrset et al. (1997)
25	*Pseudomonas elongata*	IM^l^ having carrageenan/37 °C/180 rpm/32 h/pH 7.0	37	NR^n^	7.5	NR^n^	NR^n^	Khambhaty et al. (2007a)
26	*Pseudoalteromonas* sp. AJ5-13	FMB^m^ having carrageenan/28 °C/150 rpm/36 h/pH 7.6	55	100 %/28 °C/30 min	8.0	>50 %/pH 7.2–8.6/12 h/4 °C	35 kDa	Ma et al. (2010)
27	*Pseudomonas aeruginosa* ZSL-2	MMSM^h^ having carrageenan/30 °C/170 rpm/24 h	40	NR^n^	8.0	NR^n^	NR^n^	Ziayoddin et al. (2012)
28	*Pseudoalteromonas* sp. ASY5	NR^n^	60	100 %/50 °C/7.5	7.5	100 %/pH 7–9	30 kDa	Xu et al. (2015)
29	*Tamlana* sp. HC4	FMB^m^ having carrageenan/28 °C/150 rpm/pH 7.5	30	>91 %/<45 °C/2 h	8.0	100 %/pH 7.2–8.6/4 h/30 °C	66.4 kDa	Feixue et al. (2010)
30	*Vibrio* sp. CA-1004	MM^c^ having carrageenan/25 °C/100 rpm/5 days	40	>90 %/40 °C/10 min	8.0	>80 %/pH 5–11/4 °C/24 h	35 kDa	Araki et al. (1999)
31	*Vibrio* sp. NJ-2	MM^c^ having carrageenan/30 °C/150 rpm/48 h	40	90 %/40 °C/30 min	8.0	>70 %/pH 6–10/24 h/4 °C	33 kDa	Zhu and Ning (2016)

^a^Yeast extract

^b^Weigl and Yaphe medium

^c^Minimal medium

^d^Nutrient broth

^e^Red seed powder medium

^f^Liquid medium

^g^Zobell medium

^h^Minimal mineral salts medium

^i^Bellion’s medium

^j^Basal medium

^k^Y-2 modified medium

^l^Improved medium

^m^Fermentation medium B

^n^Not reported

## Production conditions and properties

A number of bacterial species are capable of degrading carrageenan with the help of carrageenases enzyme which are mainly inducible and extracellular, however, some intracellular report are also available (Potin et al. 1995; Dyrset et al. 1997; Liu et al. 2011, 2014; Beltagy et al. 2012). It is further proposed that major and minor repeat units (dimeric units) produced from the natural polysaccharide by the action of carrageenase can act as inducer for ι-, κ-, and possibly other carrageenases (Bellion et al. 1982). Other substrates like, nutrient broth and red sea weed powder have also been practiced for the same purpose, since they offer significant benefit due to their cheaper cost and abundant availability (Li et al. 2014a, b; Kang and Kim 2015). Interestingly, it was found that various simple sugars (fructose, glucose, galactose, lactose, sucrose, mannose, maltose) did not induce carrageenase production in medium and they shows catabolite repression effect. Infact co-supplementation of simple sugars with carrageenan found to decrease the production slightly. Although the growth of bacteria was increased in the medium co-supplemented with glucose, fructose or maltose. This may be due to rapid utilization of these simple sugars by the bacterium resulting in an increase in cell mass by vigorous growth (Ziayoddin et al. 2014; George et al. 2014a, b; Sondhi et al. 2015).

The production of carrageenases are greatly influenced by nutritional and physicochemical factors, such as time, optimum temperature, optimum pH, carbon and nitrogen sources, inorganic salts, agitation and dissolved oxygen concentration (Ziayoddin et al. 2012). Various microbes require different incubation times for maximum carrageenases production. It ranges from 12 h in *Bacterium* 1 (Greer and Yaphe 1984) to 14 days in *Bacillus* sp. SYR4. (Kang and Kim 2015). Temperature and pH, giving maximum yield, correspond with the optimal conditions for the growth of the organism. Similar to other metabolic enzymes, optimum carrageenase production in bacteria is in the neutral temperature (Zhou et al. 2008; Feixue et al. 2010; Ma et al. 2013) with the exception of *Bacillus* sp. Lc50-1 which require 55 °C for production (Li et al. 2014a, b).

Carrageenases have been produced by submerged fermentation in most of the studies (Sarwar et al. 1983a, b, 1987; Youssef et al. 2012; Ziayoddin et al. 2014). However, few attempts have been made for the production of mannosidases by solid state fermentation (SSF). Ziayoddin et al. (2012) have used agro wastes for the enhanced production of carrageenases by SSF. Among them wheat bran induced the production of this enzyme more with enzyme activity of 7.44 Ug^−1^. The production of carrageenase has been increased many fold by optimization of the parameters using response surface methodology such as in case of *Cellulosimicrobium cellulans* κ-carrageenase activity to about 2.3 times higher than that obtained from the basal medium (Youssef et al. 2012) whereas in *Pseudomonas elongate*, 32-fold increase in κ-carrageenase production was achieved as compared to initial by statistical optimization method (Khambhaty et al. 2007a). Ostgaard et al. (1993) successfully grow *Pseudomonas carrageenovora* in stirred-tank fermentor (14 L) for scaled-up. Interestingly improved production (630 Uml^−1^) of the enzyme carrageenase was obtained by starting bacterial growth on a cheap carbon source (lactose) and adding small amounts of carrageenan (0.15 %) to initiate enzyme production. Similar to this Dyrset et al. (1997) showed the role of casamino acid (3.5 gl^−1^) in fed batch fermentation of *Pseudomonas carrageenovora* NUMB 302 and significantly achieved increase in enzyme activity by 2.6 times (84,000 Uml^−1^).

A comprehensive comparison of the characteristics of bacterial carrageenases (Table 2) it was observed that they can operate in a wide temperature range (25–75 °C) (Sarwar et al. 1983a, b; Mou et al. 2004; Li et al. 2014a, b). Most of them have temperature optima from 30 to 40 °C. Optimum pH for their activity varies from 5.6 to 7.5 (Khambhaty et al. 2007b; Guibet et al. 2007; Kang and Kim 2015) with carrageenases from genus of *Pseudomonas* having their optimal activity in mild alkaline conditions pH 8.0 (Ostgaard et al. 1993; Ziayoddin et al. 2012, 2014). Carrageenase from *Bacillus* sp. Lc50-1 has an optimal activity at 75 °C and a pH of 8.0 is useful for applications where high concentration of carrageenan is required because at high temperature it is more soluble as well as more oligosaccharides production (Li et al. 2014b).

Various metal ions and reagents affect carrageenase activities differently in bacteria. A common trend has been recognized, in most of the cases enzyme activity was negatively affected by heavy metals like Hg^2+^, Co^2+^, Zn^2+^, Cu^2+^, Ag^1+^, Pb^2+^ which indicated that carrageenase activity is cation independent and they were able to alter the enzyme conformation (Araki et al. 1999; Ma et al. 2010; Shangyong et al. 2013; Li et al. 2015). The inhibition by mercuric ions may indicate the importance of thiol containing amino acid residues in the carrageenase function (Li et al. 2015). However, metals like Na^+^, K^+^, Ca^2+^, Mg^2+^ are known to increase the carrageenase activity (Ma et al. 2010; Shangyong et al. 2013; Li et al. 2014b) especially Na^1+^ at high concentration (up to 500 mmol L^−1^) significantly increase the activity (Ma et al. 2013). It was hypothesized that at high concentration it changes the physical condition of carrageenan would have an effect on enzyme reaction. Nevertheless, carrageenan decomposition around the colony was visible on the carrageenan plate which contained 3 % NaCl as a component of artificial sea water medium [ASW] (Sarwar et al. 1983a, b). Reagents such as EDTA, Iodoacetic acid and Tween-80 did not affect too much to the enzyme activity (Sarwar et al. 1983a; Li et al. 2015).

Kinetics of mannan depolymerization, i.e., Michaelis–Menten constant (*K*_m_) and the maximal reaction velocity (*V*_max_) values have been reported for different bacterial carrageenases (Potin et al. 1991; Feixue et al. 2010; Ma et al. 2010). *K*_m_ and *V*_max_ values reported for *Pseudoalteromonas porphyrae* LL-1 using κ-carrageenan as a substrate were 4.4 mgml^−1^ and 0.1 mgmin^−1^ ml, respectively (Liu et al. 2011) and for *Pseudomonas elongata* MTCC 5168 were 6.7 mgml^−1^ and 4 μmol min^−1^ mg with same substrate (Khambhaty et al. 2007b). Studies on the production conditions and properties of important carrageenan degraders reported in the recent years have been summarized in Table 2.

## Cloning and expression of the carrageenase gene

Till the advent of recombinant DNA technology, enzymes were produced by fermentation of the microorganisms that express the enzymes. Purification of target enzymes from a pool of proteins requires tedious purification steps thereby increasing their costs. Recombinant DNA technology allows large scale expression of carrageenases in heterologous protein expression hosts. In recent years, a number of studies have been published on the cloning and manipulation of bacterial carrageenases genes from new and previously reported organisms with the aim of enzyme overexpression, analyzing the primary structure of the protein and protein engineering for the alteration of the enzyme properties to suit its commercial applications (Chauhan et al. 2014b, c, d, e) (Table 3).

**Table 3:** Overview of heterologously expressed carrageenase (origin, host, gene size, molecular weight, fermentation conditions, optimum temperature and stability, optimum pH and stability, family, etc.)

S. no.	Origin	Host	Gene size (bp)/enzyme (aa/kDa)	Carbon source/fermentation conditions	Temp. optima (°C) of activity	Temp. stability	pH optima of activity	pH stability	Family	Reference
1	*Alteromonas carrageenovora* ATCC 43555	*E. coli*	1191 bp/397 aa/44.4 kDa	LBM^a^/25 °C/24 h	NR^e^	NR^e^	NR^e^	NR^e^	16	Barbeyron et al. (1994)
2	*Alteromonas fortis*	*E. coli*	1425 bp/475 aa/53.3 kDa	M9 M^b^/12 °C/15 h	40	NR^e^	7.2	NR^e^	82	Michel et al. (2000); Michel et al. (2001a, b)
3	*Cellulophaga* sp. QY3	*E. coli*	1479 bp/492 aa/53.8 kDa	LBM^a^/37 °C/36 h/100 rpm	50	NR^e^	7.0	NR^e^	82	Ma et al. (2013)
4	*Cytophaga drobachiensis*	*E. coli*	1635 bp/545 aa/61.6 kDa	LBM^a^/22 °C/pH 7.2	NR	NR^e^	NR^e^	NR^e^	16	Barbeyron et al. (1998)
5	*Pseudoalteromonas carrageenovora* ATCC-43555	*E. coli*	943 bp/314 aa/105 kDa	LBM^a^ & M9 M^b^/37 °C/6 h	30	NR	7.5	NR	NR^e^	Guibet et al. (2007)
6	*Pseudoalteromonas* *carrageenovora*	*E. coli*	32.9 kDa	M9 M^b^/12 °C/15 h	NR	NR	7.0	NR	NR^e^	Michel et al. (1999)
7	*Pseudoalteromonas tetraodonis* JAM-K142	*E. coli*	1194 bp/397 aa/85 kDa (dimer)	LBM^a^/30 °C/24 h/150 rpm	30	>28.1 %/50 °C/pH 8.0/15 min	8.8	>20 %/pH 2.1–11.6/4 °C/15 min	NR^e^	Kobayashi et al. (2012)
8	*Microbulbifer thermotolerans* JAMB-A94^T^	*Bacillus subtilis*	1707 bp/569 aa/55 kDa	CLTM^c^/30 °C/72 h/250 rpm	50	NR^e^	7.5	>80 %/pH 7.0–10.0/25 °C/30 min	82	Hatada et al. (2011)
9	*Zobellia* sp. ZM-2	*E. coli*	1638 bp/545 aa/45 kDa	LBM^a^/23 °C/24 h, 120 rpm	39	>95 %/35 °C/180 min	6.0	>85 %/pH 6.0–8.0/120 min/20 °C	16	Liu et al. (2013)
10	*Zobellia galactanovorans*	*E. coli*	1473 bp/491 aa/51.9 kDa	LBMC^d^/22 °C/pH 7.2	40	NR	7.2	NR^e^	NR^e^	Barbeyron et al. (2000)

^a^Luria Bertani medium

^b^M9Medium

^c^CaCl_2_ maltose tetracycline medium

^d^Luria Bertani medium supplemented with carrageenan

^e^Not reported

The gene encoding carrageenase enzyme cloned from various bacteria has been expressed in *E. coli* in majority of the report available in literature (Guibet et al. 2007; Kobayashi et al. 2012; Liu et al. 2013). However, some genes have been expressed into other hosts also like *Bacillus subtilis* (Hatada et al. 2011).

Carrageenase production has been increased through heterologous expression in a number of cases (Michel et al. 2001b). High levels of expression has been achieved by cloning the *Microbulbifer thermotolerans* JAMB-A94^T^ carrageenase gene in the heterologous host (*Bacillus subtilis*) yielding activity of 10^5^ Ul^−1^ which is about 200 fold higher (Hatada et al. 2011). Some genetic engineering has also been done by researcher to scale up the production of carrageenase. The production of carrageenase from *Zobellia* sp. ZM-2 was increased up to 9 times using the natural signal peptide of native strain as well as removing the amino acids of about 20 kDa from C-terminal end of the gene (post translational modification) and expressing it in *E. coli* (Liu et al. 2013). In addition to this specific activity of carrageenase was also increased to some extent when they were expressed in heterologous hosts (Hatada et al. 2011; Liu et al. 2013). Cloning and expression strategies for different carrageenases have been summarized in Table 3 which might be helpful for planning future strategies for studying the carrageenases at the molecular level.

## Application of carrageenases

**Fig. 6:**
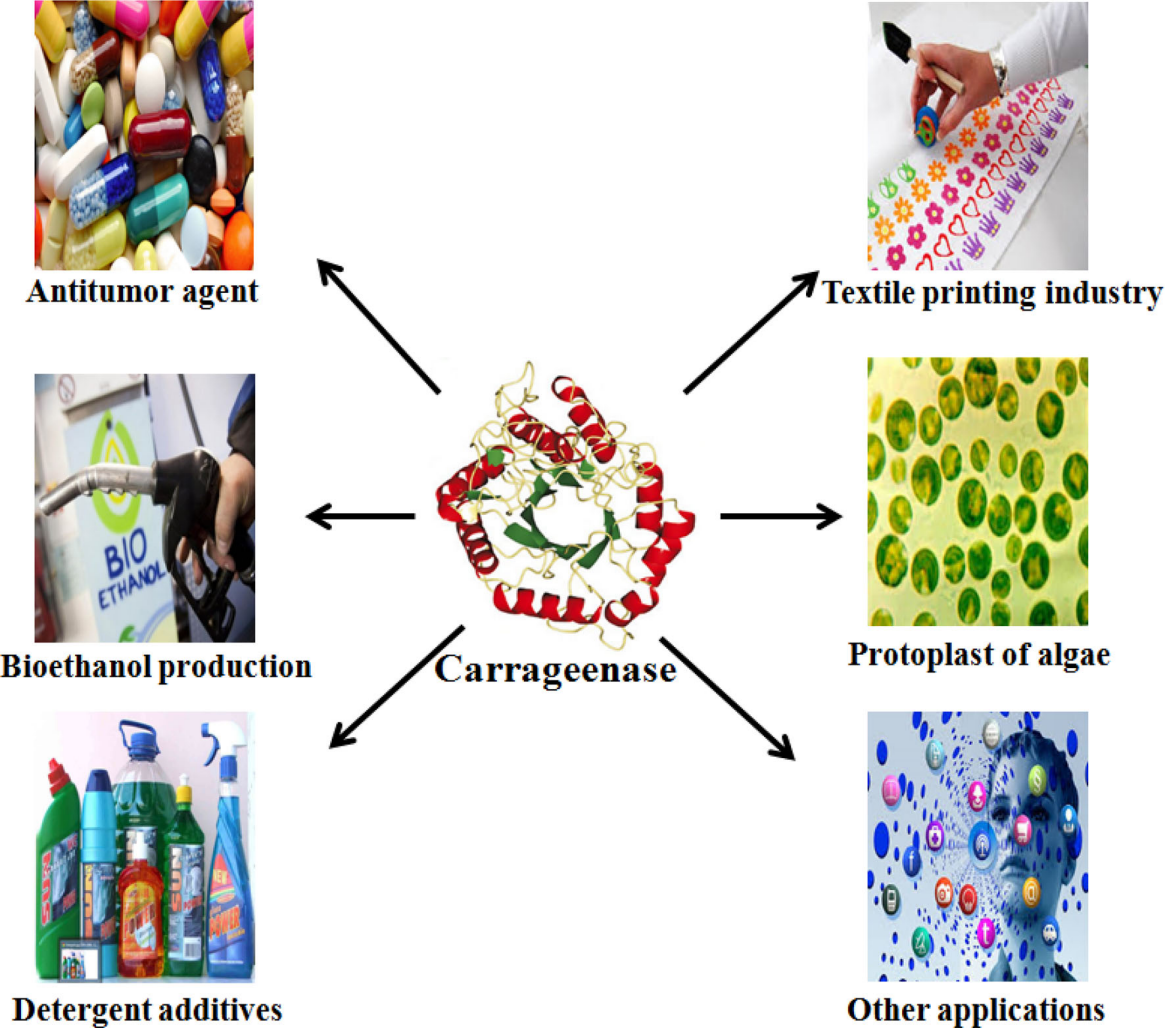
Multifarious applications of carrageenase

The broad substrate specificities of carrageenases have attracted a great deal of attention in the last decade because of their biotechnological potential in various industrial processes. The following section will discuss some of the most promising and newly explored applications of carrageenases (Fig. 6).

## In medical as antitumor agent

To improve the physical properties of polysaccharides, conversion to oligosaccharide may be the best choice. Hydrolysis products of carrageenan i.e., carrageno-oligosaccharides have potential anti-tumor properties (Yuan et al. 2006). As discussed in the section of “Enzymatic hydrolysis of carrageenan”, oligosaccharides generated by the enzymatic hydrolysis of the carrageenan are more uniform in size rather than chemical hydrolysis which further enhance their anti tumor property. It was reported that the low-molecular-weight carrageenan was effective on the anti-tumor activity, suggesting the molecular weight of oligosaccharides may play an important role (Haijin et al. 2003; Yuan and Song 2005). In addition to this bioactivity of oligosaccharides depends on several other structural features such as the degree of sulfation (DS), the sulfation position, type of sugar, and glycosidic branching. It was hypothesized that the carrageenan oligosaccharides destabilize the interaction between the glucosaminoglycan portion of proteoglycans and the extracellular matrices proteins, thus eliminating the adhesion of cancer cells to matrices, which is necessary in metastasis spread (Yuan et al. 2011).

Haijn et al. (2003) showed that carrageenan oligosaccharide with a molecular weight of 1726, administered orally at a dose of 100 mg kg^−1^ in mouse markedly inhibited tumor formation. However, the anti-tumor activity of high-sulfonated carrageenan was much less than that of the non-sulfonated or light-sulfonated preparation. The activities of the latter products on superoxide dismutase and catalase were enhanced considerably, which suggests that carrageenan oligosaccharide was effective in promoting the antioxidation ability and eliminating danger from free radicals.

## Recycling of sea weed waste into bioethanol production

Seaweeds are suitable for consumption by human beings and animals, and are a favorite food in Asian countries in particular. They are often used in fertilizers, fungicides, herbicides, and phycocolloids such as alginate, carrageenan, and agar (Kim et al. 2011). Worldwide consumption of seafood including seaweeds has increased steadily because of the associated health benefits. In recent years, the amount of seaweed waste has increased because of its use as an industrial resource and as a depolluting plant for cleaning inland sea areas and eutrophied seawater (Tang et al. 2011). Accordingly, the disposal and reuse of seaweed waste has become essential for the preservation of the marine environment and recycling of organic substances. The major species were brown seaweed and red seaweed, such as *Porphyra tenera* and *Porphyra yezoensis*. The carbohydrate content of red seaweed is 30–60 %, consisting mostly of agar and carrageenan. Unused seaweed waste is customarily discarded via landfill, incineration, or by dumping into the sea which also create environmental pollution. Kang and Kim (2015) isolated a *Bacillus* sp. SYR4 which were able to utilize sea weed waste as a carbon source by degrading both agar and carrageenan and produced reducing sugars which serves as a substrate for bioethanol production leading to 7–10 wt % of ethanol could be produced by the isolate.

## Use as a detergent additive

Carrageenans are used in dairy food products such as ice cream, yogurt, flavored milks, whipped toppings, puddings, cheeses, sour cream, juices, ready to spread icings, jams, jellies, salad dressing, candies where it use as a texture modification. In most of the food products, kappa carrageenan presents either, alone or in combination with another type of carrageenan which can cause laundry stains (Aronson et al. 2001). As carrageenans have a high affinity for cellulose fibers, therefore, they adsorb these stains to the fabric very tightly because they are not easy to remove. Polysaccharide degrading enzymes, like carrageenase, can be used as laundry additives to hydrolyze the gums present in these food stains. McDonald and Schmidt (2009) have formulated detergent composition (fabric cleaning compositions, surface cleaning compositions, oral cleaning compositions, contact lens cleaning compositions, dish cleaning compositions) containing combination of carrageenase (about 1–80 %) and surfactant (cationic, anionic or mixture thereof) which are able to hydrolyze polysaccharides efficiently hence removing the gum containing stains and giving excellent cleaning properties.

## Removal of excess printing paste after textile printing

In printing of textiles, it is common to use a printing paste containing a dye and a thickener. Among the commonly used thickeners are biological polymers (alginate, starch or modified starch, locust bean gum, galactomannan or modified galactomannan and carboxymethyl cellulose). With most printing methods, the polymer and excess dye must be removed by washing with water after the fixation of the print. Generally, a large amount of water is required for complete removal due to the high viscosity and low water solubility of the printing paste. Insufficient removal leads to unsatisfactory quality of the finished textile for the following reasons: (1) dye may be transferred to other parts of the printed textile or to other garments during laundering by the consumer. (2) Residual thickener will make printed areas stiff (Salem et al. 2008).

Pedersen et al. (1995) reported that composition comprising carrageenase along with other polysaccharide degrading enzymes will decrease process time as well as the amount of energy and water needed to achieve a satisfactory quality of the textile by hydrolyzing these polymers.

## For isolation of protoplast of algae

Carrageenases can be used for the isolation of protoplast of algae along with other cell wall degrading enzymes such as Cellulase and Macerozyme. This isolated protoplast can be used for genetic engineering experiments for the production of improved algal strains yielding better quality carrageenan of commercial value (Chen et al. 1994).

Khambhaty et al. (2007b) showed the role of bacterial carrageenase (*Pseudomonas elongata* MTCC 5168) in isolation of protoplast of red algae *Kappaphycus alvarezii*. They demonstrated that carrageenase in combination with commercially available Cellulase and Macerozyme yielded protoplast from *K. alvarezii* where in absence of κ-carrageenase, did not yield protoplasts. Morover presence or absence of individual enzymes, the composition of the osmoticum and the age of tissue were instrumental for protoplast yields (Zablackis et al. 1993).

## Other applications

### Prevent red algal bloom

The enzymes produced by marine bacteria could effectively control red algal bloom contamination. Thus, it prevents bio fouling of submerged marine surface or pipes by acting on complex polysaccharide layers (Khambhatya et al. 2007c).

### Used in structural functional studies

Carrageenases provide the opportunity to investigate the structure–function relationships of the hydrolases that degrade self-associating sulfated polysaccharides (Michel et al. 2001a).

### Protein extraction form the cell wall

Most red seaweed possesses high level of proteins (10–30 % dry weight) (Morgan et al. 1980). These proteins can be extracted by hydrolytic enzymes like carrageenase (Fleurence et al. 1995). For example, the degradation of cell Wall polysaccharides by hydrolytic enzymes is used for the isolation of extensin, a protein linked to cell wall polysaccharide of higher plants (Lamport 1969).

## Conclusion

Microbial carrageenases have attracted great attention in near past because of their useful applications. Such enzyme systems are not only of academic interest since they have potential biotech applications in a wide range of industrial enzyme markets. Exploitation of biodiversity to provide microorganisms that produce carrageenases well suited for their diverse applications is considered to be one of the most promising future alternatives. The knowledge summarized in this review, regarding the known sources of carrageenases, and their properties would be a great help to study these enzymes, so that they can be effectively utilized for various biotechnological processes.
